# Associations between Mitral Annular and Left Atrial Volume Changes in Healthy Adults–Detailed Analysis from the Three-Dimensional Speckle-Tracking Echocardiographic MAGYAR-Healthy Study

**DOI:** 10.31083/j.rcm2306194

**Published:** 2022-05-27

**Authors:** Attila Nemes, Árpád Kormányos, Nóra Ambrus, Csaba Lengyel

**Affiliations:** ^1^Department of Medicine, Albert Szent-Györgyi Medical School, University of Szeged, 6725 Szeged, Hungary

**Keywords:** healthy, mitral annulus, left atrium, three-dimensional, echocardiography

## Abstract

**Background:**

Three-dimensional (3D) speckle-tracking echocardiography (3DSTE) is one of the newest development in non-invasive imaging offering simultaneous 3D evaluation of atria and valvular annuli. 3DSTE was used to analyze correlations between left atrial (LA) volume changes and mitral annular (MA) dimensions and functional properties in healthy adult subjects.

**Methods:**

A total of 297 healthy subjects were enrolled in this retrospective cohort study, from which insufficient quality of images was responsible for the exclusion of 98 cases (33%). The remaining study population consisted of 199 healthy adults without valvular regurgitation/stenosis in sinus rhythm (mean age: 33.5 ± 12.7 years, 104 males, body mass index: 24.7 ± 1.2 kg/$m^{2}$, systolic and diastolic blood pressure: 118.2 ± 3.4 mmHg and 78.3 ± 4.5 mmHg, respectively). Two-dimensional Doppler echocardiography and 3DSTE were performed in all cases.

**Results:**

Larger LA volumes were associated with more dilated MA dimensions with its reduced function. Elevated LA stroke volumes could be demonstrated only in systole and end-diastole, while increased LA emptying fraction was present only in end-diastole. Reduced MA fractional area change was associated with larger diastolic LA volumes, smaller early diastolic LA stroke volume, in addition all LA emptying fractions were smaller as well. Correlations could be demonstrated between LA and MA parameters.

**Conclusions:**

3DSTE is suitable not only for chamber quantifications, but also for the assessment of valvular annular dimensions. Strong relationship exists between LA volumes and MA dimensions and functional properties.

## 1. Background

Both left atrium (LA) and mitral annulus (MA) have cyclic changes in dimensions during the heart cycle [1, 2]. Three-dimensional (3D) speckle-tracking echocardiography (3DSTE) is one of the newest development in cardiac imaging offering simultaneous 3D examination of atria and valvular annuli [3, 4, 5]. There are limited number of studies assessing physiologic connections between LA and MA in healthy subjects, therefore each study assessing their relationship could increase our knowledge in this field [2, 6]. Therefore, in the present study, correlations between MA and LA volume changes were analyzed by 3DSTE in healthy adults.

## 2. Materials and Methodologies

### 2.1 Subjects

Hundreds of healthy adult volunteers were recruited as part of the screening between 2011–2015 and were examined at the outpatient cardiology clinic at the University of Szeged, Hungary. Screening involved physical examination, standard 12-lead electrocardiography (ECG), two-dimensional Doppler echocardiography (2DE) and 3DSTE. This retrospective cohort study comprised 297 healthy cases, from which insufficient quality of images was responsible for the exclusion of 98 cases (33%). The remaining study population consisted of 199 healthy adults in sinus rhythm (mean age: 33.5 ± 12.7 years, 104 males, body mass index: 24.7 ± 1.2 kg/$m^{2}$, systolic and diastolic blood pressure: 118.2 ± 3.4 mmHg and 78.3 ± 4.5 mmHg, respectively). All subjects with symptoms, risk factors, pathological states or known disorders and who use of medicinal products were excluded. Moreover, physiological examination, laboratory findings, ECG and echocardiography proved to be normal in all cases. All echocardiographic studies and data acquisition and analysis for 3DSTE were performed by the same observer (ÁK) between 2012 and 2015. The present study serves as a part of the MAGYAR-Healthy Study (Motion Analysis of the heart and Great vessels bY three-dimensionAl speckle-tRacking echocardiography in Healthy subjects). This study aimed to determine physiologic relationships between 3DSTE-derived parameters in healthy adults among others (‘magyar’ means ‘Hungarian’ in Hungarian language). The study was approved by the Institutional and Regional Human Biomedical Research Committee of University of Szeged and all the participants provided a written informed consent.

### 2.2 2DE

All the 2DE images were acquired by a Toshiba ${\mathrm{Artida}}^{\text{TM}}$ echocardiographic machine (Toshiba Medical Systems, Tokyo, Japan) with a PST-30SBP phased-array transducer (Toshiba Medical Systems, Tokyo, Japan) according to the guidelines [7]. Results showed normal findings in all cases. Absence of significant valvular stenoses and ≥grade 1 valvular regurgitations was confirmed by Doppler ultrasound method. Diastolic function was characterized by Doppler-derived early (E) and late (A) diastolic transmitral inflow velocities and their ratio (E/A).

### 2.3 3DSTE

As a first step, separate LA- and left ventricle (LV)-focused 3D echocardiographic datasets were acquired digitally by the same Toshiba ${\mathrm{Artida}}^{\text{TM}}$ echocardiographic machine using a PST-25SX matrix-array transducer (Toshiba Medical Systems, Tokyo, Japan). Data acquisitions were done during breath-holding from the apical position in sinus rhythm [4, 5]. For better image quality, six smaller wedge-shaped subvolumes were acquired focused on LA/LV within six cardiac cycles from which a pyramidal (also called as full volume) dataset was created by the software automatically. As a second step, detailed analysis was performed offline by using 3D Wall Motion Tracking software version 2.7 (Toshiba Medical Systems, Tokyo, Japan).

### 2.4 LA Evaluations

**Fig. 1.**
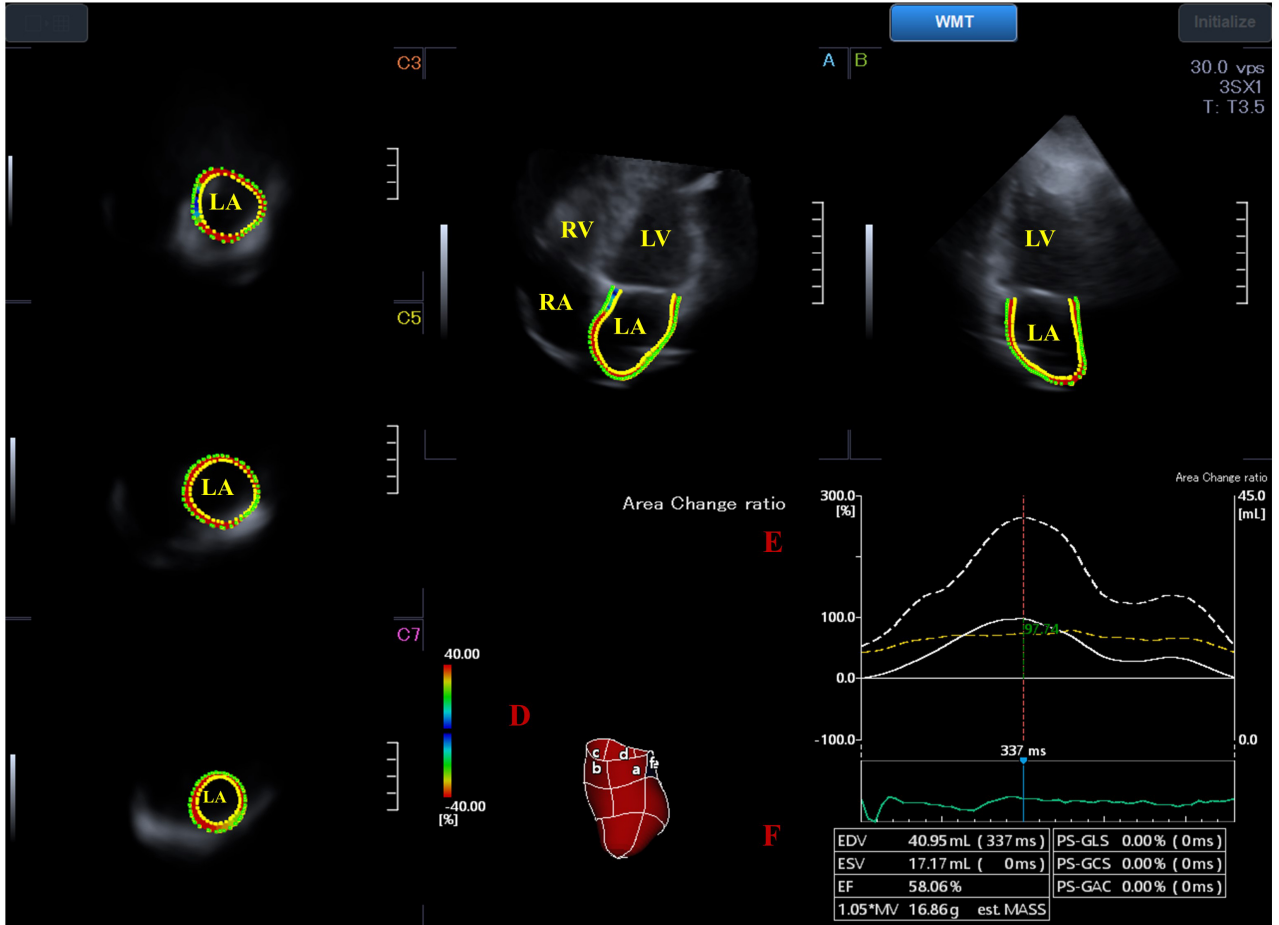
**Three-dimensional (3D) speckle tracking echocardiography-derived full-volume dataset from which the 3D cast of the left atrium (LA) is extracted in a healthy subject**. (A) Apical four-chamber view. (B) Apical two-chamber view. (C3) Short-axis view at basal LA level, (C5) short-axis view at midatrial LA level and (C7) short-axis view at superior LA level are presented (D) with a 3D virtual model of the LA. (E) Time–global LA volume change (dashed line) and time–global LA area change ratio (strain) curves (white line) with (F) calculated volumetric LA data are also shown. Abbreviations: EDV, LA volume at end-diastole; ESV, LA volume at end-systole; EF, LA ejection fraction; LA, left atrium; LV, left ventricle; RA, right atrium; RV, right ventricle.

Following optimalisations on LA-focused images, apical two- (AP2CH) and AP4CH views and 3 short-axis views at basal, midatrial and superior LA levels at end-diastole helped creation of virtual 3D cast of the LA, from which LA volumes were calculated (Fig. 1) [8]:

- maximum LA volume ($V_{\text{max}}$) obtained at mitral valve opening in the end-systolic frame,

- LA volume at the onset of atrial systole ($V_{\text{preA}}$) obtained before mitral valve reopening in the last frame or at the p wave on ECG,

- minimum LA volume ($V_{\text{min}}$) obtained at mitral valve closure in the end-diastolic frame.

The following LA functional properties were calculated:

- LA total stroke (emptying) volume (TASV) = ($V_{\text{max}}$ – $V_{\text{min}}$) *(*s*ystolic reservoir function)*

- LA total emptying fraction (TAEF) = ${\mathrm{TASV/V}}_{\text{max}}$* (*s*ystolic reservoir function)*

- LA passive stroke (emptying) volume (PASV) = $V_{\text{max}}$ – $V_{\text{preA}}$* (early diastolic conduit function)*

- LA passive emptying fraction (PAEF) = ${\mathrm{PASV/V}}_{\text{max}}$* (early diastolic conduit function)*

- LA active stroke (emptying) volume (AASV) = $V_{\text{preA}}$ – $V_{\text{min}}$* (late diastolic booster pump function, active contraction phase)*

- LA active emptying fraction (AAEF) = ${\mathrm{AASV/V}}_{\text{preA}}$* (late diastolic booster pump function, active contraction phase)*

Healthy subjects were classified into 3 groups according to the normal maximum end-systolic LA volume as presented in a recent study. Estimated mean ± SD served as the lower (30 mL) and upper (50 mL) values [9].

### 2.5 MA Quantifications 

**Fig. 2.**
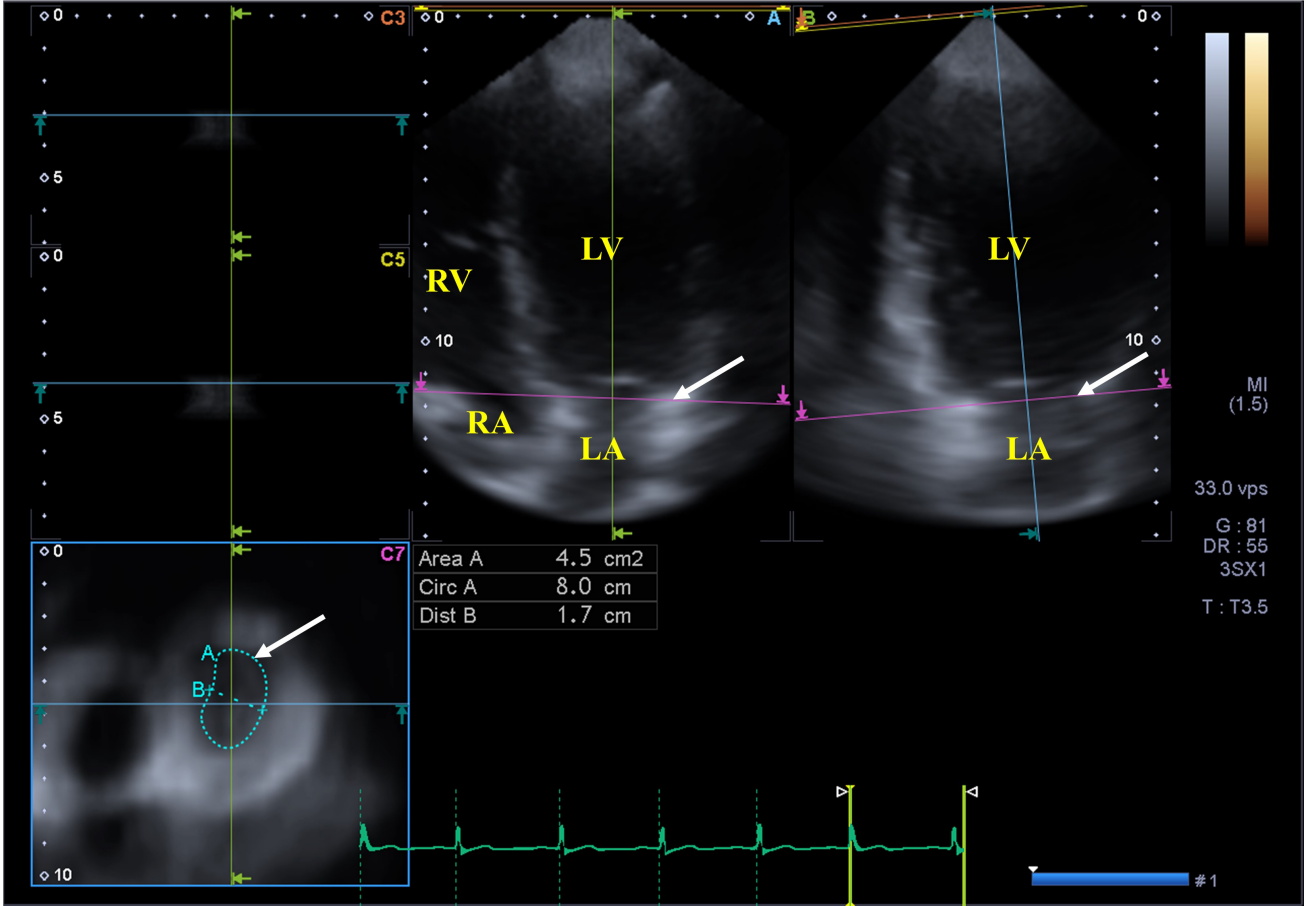
**Three-dimensional speckle tracking echocardiography-derived three-dimensional dataset from which the mitral annulus is extracted**. (A) Apical four-chamber view, (B) apical two-chamber view and a cross sectional view at the level of the mitral annulus (C7) following optimizations in apical four- and two-chamber views. The mitral annular plane is shown by a white arrow on long- (A, B) and short-axis (C7) images. Abbreviations: LA, left atrium; LV, left ventricle; RA, right atrium; RV, right ventricle; Area, MA area, Circ, MA perimeter, Dist, MA diameter.

Using the LV-focused datasets, image planes were optimized on lateral and septal MA-LV edges/endpoints on AP2CH and AP4CH views [6, 10]. MA dimensions were assessed on the C7 short-axis view as demonstrated on Fig. 2. Several MA parameters featuring 2D-projected MA dimensions were measured with respect to the cardiac cycle [11]:

- MA diameter (MAD) was measured as a perpendicular line drawn from the peak of the MA curvature to the middle of the straight MA border, and it was measured both at end-diastole (MAD-D) and end-systole (MAD-S),

- MA area (MAA) was measured by planimetry both at end-diastole (MAA-D) and end-systole (MAA-S),

- MA perimeter (MAP) was measured by planimetry both at end-diastole (MAP-D) and end-systole (MAP-S).

MA diameter and MA area data were used for calculation of MA functional properties:

- MA fractional shortening (MAFS) was measured as [MAD-D – MAD-S]/MAD-D × 100

- MA fractional area change (MAFAC) was measured as [MAA-D – MAA-S]/MAA-D × 100.

### 2.6 Statistical Analysis

Categorical variables were presented as absolute values (percentages) and continuous variables were presented as mean ± standard deviation. *p* values < 0.05 were considered significant in statistical analyses. All tests were two-sided. Shapiro-Wilks test was used for the evaluation of normality of distribution. To assess homogeneity of variances, Levene’s test was used. If distribution of dataset proved to be normal, Student’s *t*-test was applied, while if distribution of dataset was not normal, Mann-Whitney-Wilcoxon test was used. Fisher’s exact test was applied to compare categorical variables. Receiver operator curves (ROC) were constructed to define prognostic impact of MAFAC. Bland–Altman method was used for intraobserver and interobserver agreements together with calculation of Pearson’s correlation coefficients in 25 healthy adults. RStudio (RStudio Team (2015) RStudio: Integrated Development for R. RStudio, Inc., Boston, MA, USA) and MATLAB version 8.6 software package (The MathWorks Inc., Natick, MA, USA, 2015) were used for statistical and data analyses.

## 3. Results

### 3.1 Routine Two-Dimensional Echocardiographic Data

LV end-diastolic and end-systolic diameters (48.4 ± 3.7 mm and 39.1 ± 23.5 mm, respectively) and LV end-diastolic and end-systolic volumes (108.7 ± 26.1 mL and 37.2 ± 9.9 mL, respectively), thickness of the interventricular septum and posterior wall (8.97 ± 1.48 mm and 9.16 ± 1.57 mm, respectively) and LV ejection fraction (65.7 ± 4.6%) were calculated in all subjects. No subject showed ≥grade 1 valvular insufficiency or stenosis. E/A proved to be 1.33 ± 0.31 cm/s.

### 3.2 3DSTE-Derived LA and MA Data

LA and MA features measured by 3DSTE are presented in Table 1. The subject group was divided into three subgroups according to their $V_{\text{max}}$ using 30 mL and 50 mL as cut-offs. Higher end-systolic $V_{\text{max}}$ was associated with elevated diastolic $V_{\text{min}}$ and $V_{\text{preA}}$ and TASV, PASV and AASV and their body surface area-indexed counterpart. When LA emptying fractions were examined, only TAEF was found to be significantly decreased when $V_{\text{max}}$ >50 mL, TAEF and AAEF did not change with increasing $V_{\text{max}}$. When MA dimensions were analyzed according to the increasing $V_{\text{max}}$, dilated MA could be measured in subjects with larger $V_{\text{max}}$ (between 30 mL and 50 mL) regardless in which cardiac phase it was measured. Further dilation could be detected only in MAD-S, MAA-S and MAP-S in case of $V_{\text{max}}$ >50 mL (Table 2). Enlarged $V_{\text{max}}$ was associated with the decrease of MAFAC.

**Table 1.** **Three-dimensional speckle-tracking echocardiography-derived left atrial and mitral annular data of healthy subjects**.

	Parameters	Measures
Left atrial volumes		
	Maximum LA volume ($V_{\text{max}}$) (mL)	40.9 ± 13.2
	Maximum LA volume-indexed (mL)	22.2 ± 7.9
	Pre-atrial contraction LA volume ($V_{\text{preA}}$) (mL)	27.9 ± 12.0
	Pre-atrial contraction LA volume-indexed (mL)	15.2 ± 7.2
	Minimum LA volume ($V_{\text{min}}$) (mL)	19.6 ± 8.5
	Minimum LA volume-indexed (mL)	10.7 ± 5.1
Left atrial stroke volumes		
	Total atrial stroke volume (TASV) (mL)	21.3 ± 8.0
	Total atrial stroke volume-indexed (mL)	11.6 ± 4.8
	Passive atrial stroke volume (PASV) (mL)	13.0 ± 5.7
	Passive atrial stroke volume-indexed (mL)	7.1 ± 3.4
	Active atrial stroke volume (AASV) (mL)	8.3 ± 5.6
	Active atrial stroke volume-indexed (mL)	4.5 ± 3.4
Let atrial emptying fractions		
	Total atrial emptying fraction (TAEF) (%)	52.3 ± 12.0
	Passive atrial emptying fraction (PAEF) (%)	32.8 ± 12.8
	Active atrial emptying fraction (AAEF) (%)	29.0 ± 11.7
Mitral annular end-diastolic data		
	Mitral annular diameter (MAD-D) (mm)	2.42 ± 0.43
	Mitral annular area (MAA-D) (${\mathrm{mm}}^{2}$)	7.25 ± 2.25
	Mitral annular perimeter (MAP-D) (mm)	10.18 ± 1.54
Mitral annular end-systolic data		
	Mitral annular diameter (MAD-S) (mm)	1.58 ± 0.39
	Mitral annular area (MAA-S) (${\mathrm{mm}}^{2}$)	3.41 ± 1.24
	Mitral annular perimeter (MAP-S) (mm)	7.06 ± 1.25
Mitral annular functional properties		
	Mitral annular fractional area change (MAFAC) (%)	51.5 ± 15.4
	Mitral annular fractional shortening (MAFS) (%)	33.9 ± 15.2

Abbreviations: LA, left atrial; D, end-diastolic; S, end-systolic.

**Table 2.** **Dependence of mitral and left atrial dimensions and functional properties on the maximum left atrial volume**.

	$V_{\text{max}}$ < 30 mL	30 mL ≤ $V_{\text{max}}$ ≤ 50 mL	$V_{\text{max}}$ >50 mL
	(n = 42)	(n = 120)	(n = 37)
$V_{\text{max}}$ (mL)	25.4 ± 3.7	39.6 ± 5.6*	62.0 ± 9.3*†
$V_{\text{max}}$-indexed (mL)	14.9 ± 2.4	21.8 ± 3.4*	33.1 ± 5.7*†
$V_{\text{preA}}$ (mL)	16.8 ± 4.3	26.3 ± 6.9*	45.4 ± 11.7*†
$V_{\text{preA}}$-indexed (mL)	9.4 ± 2.5	14.5 ± 4.1*	24.1 ± 7.0*†
$V_{\text{min}}$ (mL)	12.2 ± 4.0	18.7 ± 5.4*	30.5 ± 9.2*†
$V_{\text{min}}$-indexed (mL)	7.1 ± 2.6	10.2 ± 3.2*	16.2 ± 5.4*†
TASV (mL)	13.3 ± 4.3	20.8 ± 5.1*	31.5 ± 7.3*†
TASV-indexed (mL)	7.7 ± 2.6	11.5 ± 3.1*	16.9 ± 4.3*†
PASV (mL)	8.6 ± 3.6	13.3 ± 4.7*	16.6 ± 7.1*†
PASV-indexed (mL)	5.2 ± 2.2	7.2 ± 2.8*	8.9 ± 4.4*†
AASV (mL)	4.7 ± 3.0	7.6 ± 3.5*	14.9 ± 7.6*†
AASV-indexed (mL)	2.7 ± 1.8	4.3 ± 2.1*	7.9 ± 4.6*†
TAEF (%)	52.1 ± 14.2	52.8 ± 11.4	51.2 ± 10.8
PAEF (%)	33.9 ± 13.1	34.0 ± 12.4	27.3 ± 12.1*†
AAEF (%)	27.4 ± 14.6	28.6 ± 10.2	32.3 ± 10.1
MAD-D (cm)	2.26 ± 0.37	2.45 ± 0.45*	2.53 ± 0.40*
MAA-D (${\mathrm{cm}}^{2}$)	6.37 ± 1.73	7.40 ± 2.30*	7.75 ± 2.45*
MAP-D (cm)	9.61 ± 1.30	10.26 ± 1.53*	10.59 ± 1.66*
MAD-S (cm)	1.44 ± 0.34	1.58 ± 0.38*	1.73 ± 0.38*†
MAA-S (${\mathrm{cm}}^{2}$)	2.79 ± 0.86	3.43 ± 1.25*	4.02 ± 1.24*†
MAP-S (cm)	6.44 ± 0.96	7.09 ± 1.27*	7.62 ± 1.17*†
MAFAC (%)	53.8 ± 16.0	52.3 ± 14.7	45.8 ± 15.5*†
MAFS (%)	35.5 ± 15.3	34.4 ± 15.4	31.0 ± 13.9

Abbreviations: AAEF, active atrial emptying fraction; AASV, active atrial stroke volume; PAEF, passive atrial emptying fraction; PASV, passive atrial stroke volume; TAEF, total atrial emptying fraction; TASV, total atrial stroke volume; $V_{\text{max}}$, maximum left atrial volume; $V_{\text{min}}$, minimum left atrial volume; $V_{\text{preA}}$, pre-atrial contraction left atrial volume; MAD, mitral annular diameter; MAA, mitral annular area; MAP, mitral annular perimeter; MAFAC, mitral annular fractional area change; MAFS, mitral annular fractional shortening; D, end-diastolic; S, end-systolic. *p < 0.05 vs. $V_{\text{max}}$ <30 mL. †*p *< 0.05 vs. 30 mL ≤ $V_{\text{max}}$ ≤ 50 mL.

**Fig. 3.**
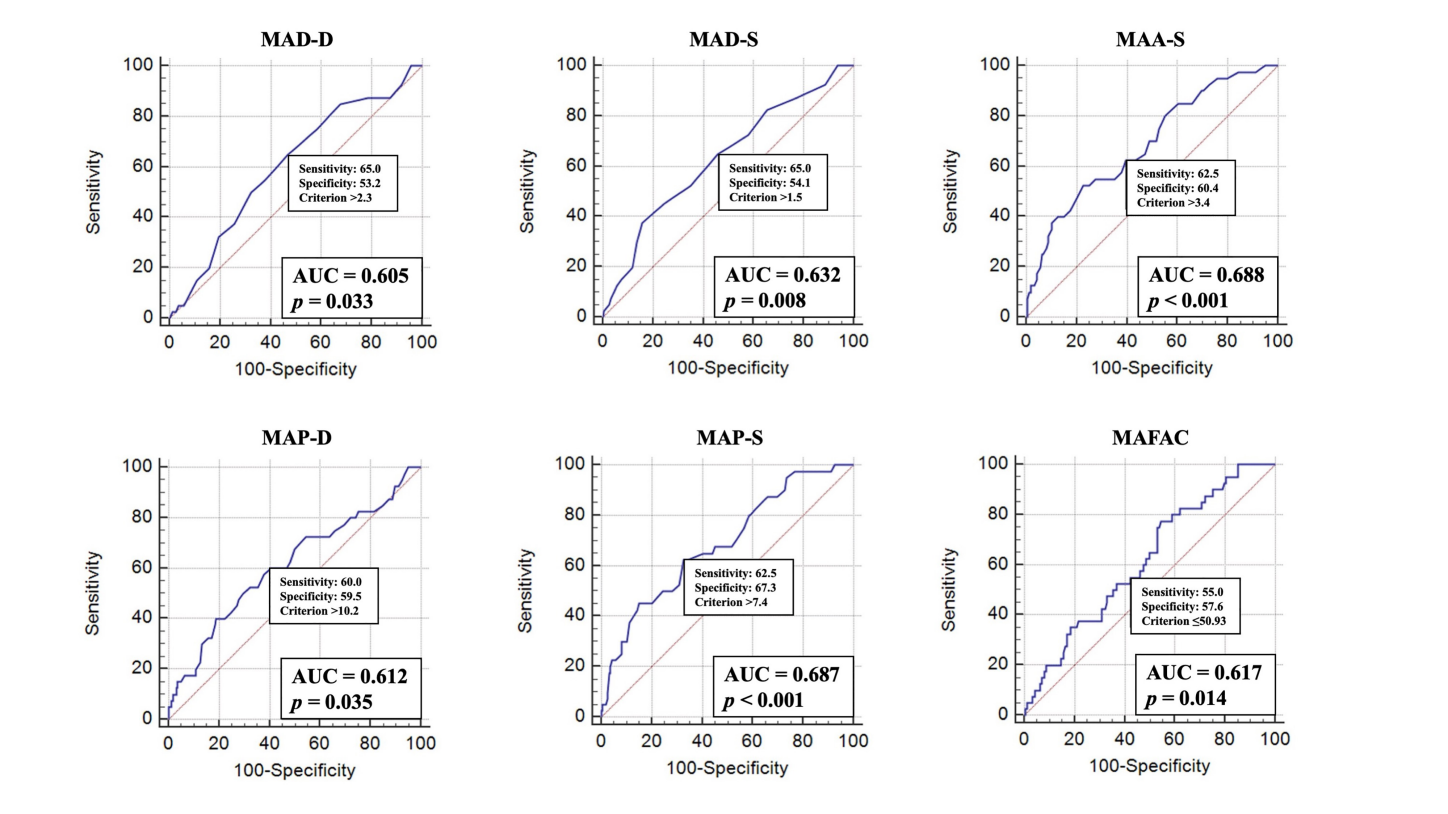
**Prognostic value of mitral annular dimensions and mitral annular fractional area change to predict larger than 50 mL maximum left atrial volume**. Abbreviations: MAD-D, end-diastolic mitral annular diameter; MAD-S, end-systolic mitral annular diameter; MAA-S, end-systolic mitral annular area; MAP-D, end-diastolic mitral annular perimeter; MAP-S, end-systolic mitral annular perimeter; MAFAC, mitral annular fractional area change; AUC, area under curve.

Some MA morphological parameters and MAFAC were found to be associated with $V_{\text{max}}$ >50 mL. The cut-offs for MAD-D, MAD-S, MAA-S, MAP-D, MAP-S and MAFAC proved to be 2.3 cm (Area under curve, AUC) = 0.605, *p* = 0.033), 1.5 cm (AUC = 0.632, *p* = 0.008), 3.4 ${\mathrm{cm}}^{2}$ (AUC = 0.688, *p *< 0.001), 10.2 cm (AUC = 0.612, *p* = 0.035), 7.4 cm (AUC = 0.687, *p *< 0.001) and 50.93% (AUC 0.617, *p* = 0.014), respectively (Fig. 3).

All LA volumes were elevated with dilated MAD-D and MAD-S, only increased $V_{\text{preA}}$ and $V_{\text{min}}$ and their counterpart were accompanied with reduced MAFAC. Elevated TASV and AASV and their indexed counterpart were found when MAD-D proved to be dilated, similar findings were not found with MAD-S. PASV and indexed counterpart were found to be elevated in subject with increased MAFAC. When dilation of MAD-D and MAD-S and their association with emptying fractions were examined, dilated MAD-D was accompanied with elevated AAEF and dilated MAD-S was associated with decreased TAEF and PAEF. Elevated MAFAC was associated with increased TAEF, PAEF and AAEF (Table 2). All end-systolic and end-diastolic MA dimensions showed dilation with dilated MAD-D and MAD-S. Elevated MAFAC was accompanied with dilated MAD-D and decreased MAD-S (Table 3). No correlations were found between E/A and other parameters.

**Table 3.** **Dependence of mitral and left atrial dimensions and functional properties on mitral annular diameters and fractional area change**.

	MAD-D ≤2.3 cm	MAD-D >2.3 cm	MAD-S ≤1.5 cm	MAD-S >1.5 cm	MAFAC ≤50.93%	MAFAC >50.93%
	(n = 76)	(n = 123)	(n = 101)	(n = 98)	(n = 88)	(n = 111)
$V_{\text{max}}$ (mL)	37.7 ± 12.9	42.7 ± 13.1*	38.3 ± 13.1	43.5 ± 13.0†	42.3 ± 13.9	39.7 ± 12.5
$V_{\text{max}}$-indexed (mL)	21.9 ± 7.9	23.0 ± 7.8*	21.2 ± 8.1	23.5 ± 8.0†	23.5 ± 8.5	22.3 ± 7.6
$V_{\text{preA}}$ (mL)	25.5 ± 12.0	29.4 ± 11.8*	24.8 ± 11.6	31.1 ± 11.7†	30.3 ± 12.7	26.1 ± 11.1‡
$V_{\text{preA}}$-indexed (mL)	14.2 ± 7.4	15.9 ± 7.2*	13.8 ± 7.1	16.8 ± 7.2†	16.8 ± 7.7	14.2 ± 6.7‡
$V_{\text{min}}$ (mL)	18.5 ± 8.4	20.2 ± 8.5*	16.8 ± 7.4	22.5 ± 8.7†	22.0 ± 9.3	17.7 ± 7.2‡
$V_{\text{min}}$-indexed (mL)	11.3 ± 5.2	10.9 ± 5.2*	9.3 ± 4.7	12.3 ± 5.3†	12.0 ± 5.7	9.5 ± 4.1‡
TASV (mL)	19.2 ± 7.7	22.5 ± 7.8*	21.5 ± 8.8	21.1 ± 7.1	20.3 ± 7.7	22.1 ± 8.1
TASV-indexed (mL)	10.6 ± 4.6	12.2 ± 4.7*	11.6 ± 5.3	11.2 ± 4.4	11.3 ± 5.7	11.5 ± 4.8
PASV (mL)	12.3 ± 5.3	13.4 ± 5.9	13.5 ± 5.8	12.4 ± 5.6	12.1 ± 5.7	13.6 ± 5.6‡
PASV-indexed (mL)	6.6 ± 3.3	7.3 ± 3.7	7.6 ± 3.6	6.7 ± 3.4	6.8 ± 3.5	7.3 ± 3.5‡
AASV (mL)	7.0 ± 5.6	9.1 ± 5.5*	8.0 ± 6.3	8.7 ± 4.8	8.2 ± 5.1	8.5 ± 6.0
AASV-indexed (mL)	3.8 ± 3.4	4.9 ± 3.3*	4.4 ± 3.8	4.6 ± 2.9	4.6 ± 3.1	4.7 ± 3.5
TAEF (%)	51.3 ± 12.4	53.0 ± 11.6	55.8 ± 12.0	48.8 ± 11.0†	48.3 ± 12.1	55.5 ± 10.9‡
PAEF (%)	33.8 ± 13.1	32.2 ± 12.5	36.3 ± 12.8	29.3 ± 11.9†	29.4 ± 13.1	35.3 ± 12.1‡
AAEF (%)	26.2 ± 11.2	30.7 ± 11.7*	30.5 ± 12.6	27.4 ± 10.6	26.5 ± 10.9	31.0 ± 11.9‡
MAD-D (cm)	2.00 ± 0.18	2.67 ± 0.33*	2.27 ± 0.37	2.56 ± 0.44†	2.35 ± 0.41	2.47 ± 0.43‡
MAA-D (${\mathrm{cm}}^{2}$)	5.48 ± 1.23	8.34 ± 2.02*	6.49 ± 1.91	8.02 ± 2.33†	6.60 ± 1.97	7.76 ± 2.32‡
MAP-D (cm)	9.10 ± 1.14	10.85 ± 1.35*	9.73 ± 1.40	10.65 ± 1.54†	9.74 ± 1.36	10.54 ± 1.57‡
MAD-S (cm)	1.40 ± 0.28	1.70 ± 0.40*	1.28 ± 0.19	1.89 ± 0.28†	1.77 ± 0.36	1.44 ± 0.35‡
MAA-S (${\mathrm{cm}}^{2}$)	2.74 ± 0.81	3.83 ± 1.27*	2.56 ± 0.67	4.27 ± 1.09†	4.09 ± 1.19	2.88 ± 1.01‡
MAP-S (cm)	6.43 ± 1.02	7.45 ± 1.22*	6.30 ± 1.00	7.82 ± 1.01†	7.71 ± 1.10	6.55 ± 1.13‡
MAFAC (%)	48.3 ± 16.2	53.3 ± 14.5*	58.2 ± 13.9	44.6 ± 13.8†	37.3 ± 10.1	62.8 ± 7.5‡
MAFS (%)	29.9 ± 14.7	36.2 ± 15.5*	42.6 ± 11.4	25.1 ± 13.5†	24.3 ± 12.6	41.6 ± 12.5‡

Abbreviations: AAEF, active atrial emptying fraction; AASV, active atrial stroke volume; PAEF, passive atrial emptying fraction; PASV, passive atrial stroke volume; TAEF, total atrial emptying fraction; TASV, total atrial stroke volume; $V_{\text{max}}$, maximum left atrial volume; $V_{\text{min}}$, minimum left atrial volume; $V_{\text{preA}}$, pre-atrial contraction left atrial volume; MAD, mitral annular diameter; MAA, mitral annular area; MAP, mitral annular perimeter; MAFAC, mitral annular fractional area change; MAFS, mitral annular fractional shortening; D, end-diastolic; S, end-systolic. *p < 0.05 vs. MAD-D ≤2.3 cm. †*p *< 0.05 vs. MAD-S ≤1.5 cm. ‡*p *< 0.05 vs. MAFAC ≤50.93%.

### 3.3 Reproducibility Measurements 

The average ± standard deviation difference in parameters measured 2 times by same examiner and two examiners for the assessment of 3DSTE-derived LA and MA features are shown in Table 4.

**Table 4.** **Intraobserver and interobserver variability for three-dimensional speckle-tracking echocardiography-derived left atrial volumetric parameters and mitral annular dimensions**.

	Intraobserver agreement		Interobserver agreement	
	Average ± standard deviation difference in parameters measured 2 times by same examiner	Pearson’s coefficient	Average ± standard deviation difference in parameters measured by 2 examiners	Pearson’s coefficient
$V_{\text{max}}$	0.4 ± 3.8 mL	0.95 (*p *< 0.001)	0.5 ± 5.3 mL	0.97 (*p <* 0.001)
$V_{\text{preA}}$	0.4 ± 3.1 mL	0.96 (*p *< 0.001))	0.3 ± 4.1 mL	0.97 (*p <* 0.001)
$V_{\text{min}}$	–1.0 ± 5.8 mL	0.88 (*p *< 0.001))	–0.9 ± 4.8 mL	0.86 (*p *< 0.001)
MAD-D	0.00 ± 0.21 mm	0.95 (*p *< 0.001)	0.02 ± 0.20 mm	0.96 (*p <* 0.001)
MAA-D	0.02 ± 0.89 ${\mathrm{mm}}^{2}$	0.96 (*p *< 0.001)	0.00 ± 0.67 ${\mathrm{mm}}^{2}$	0.97 (*p *< 0.001)
MAP-D	–0.07 ± 0.91 mm	0.93 (*p *< 0.001)	–0.10 ± 0.88 mm	0.92 (*p *< 0.001)
MAD-S	0.00 ± 0.22 mm	0.96 (*p *< 0.001)	0.00 ± 0.22 mm	0.97 (*p *< 0.001)
MAA-S	0.00 ± 0.29 ${\mathrm{mm}}^{2}$	0.98 (*p *< 0.001)	–0.01 ± 0.37 ${\mathrm{mm}}^{2}$	0.97 (*p *< 0.001)
MAP-S	0.06 ± 0.63 mm	0.98 (*p *< 0.001)	0.03 ± 0.51 mm	0.98 (*p *< 0.001)

Abbreviations: $V_{\text{max}}$, maximum left atrial volume; $V_{\text{min}}$, minimum left atrial volume; $V_{\text{preA}}$, pre-atrial contraction left atrial volume; MAD, mitral annular diameter; MAA, mitral annular area; MAP, mitral annular perimeter; D, end-diastolic; S, end-systolic.

## 4. Discussion

In a recent work, correlations could be confirmed between LA volumes and volume-based functional properties and MA dimensions and calculated functional parameters in healthy adults [6]. While $V_{\text{max}}$ correlated with both systolic and diastolic MA parameters, diastolic $V_{\text{min}}$ and $V_{\text{preA}}$ showed correlations only with systolic MA parameters. While systolic TASV correlated with both systolic and diastolic MA parameters, diastolic PASV showed correlations only with diastolic MA parameters. Neither AASV nor any emptying fractions showed correlations with any MA morphological or functional parameters. When LA volumetric parameters were examined they did not correlate with MAFS and MAFAC, but systolic TASV and diastolic PASV showed correlations with MAFS [6].

The present study aimed to provide further detailed exploration of the relationship between MA and LA volume changes in healthy adults by 3DSTE. Larger LA was found to be associated with more dilated MA dimensions and its reduced function in otherwise healthy subjects without mitral regurgitation. Moreover, it was also verified that dilated MA was associated with dilated LA volumes with respect to the cardiac cycle. Interestingly, elevated LA stroke volumes could be detected only in systole and end-diastole, while increased LA emptying fraction was present only in end-diastole. Reduced MA fractional area change was associated with larger diastolic LA volumes, smaller early diastolic LA stroke volume and all LA emptying fractions were reduced as well. These results suggest fine cooperation between LA volumes and volume-based functional properties and MA dimensions even in healthy subjects.

The LA is located on the left side of the heart and is connected to the LV via the MA. The LA has a dynamic motion with respect to the heart cycle working as a reservoir during LV systole, being a conduit during early diastole forwarding blood to the LV from the pulmonary veins and an actively contracting pump during late diastole [1]. The saddle-shaped left-sided atrio-ventricular mitral valve includes MA, leaflets, papillary muscles and chordae [12]. Although MA is a fibrous ring, it is affected by the contractility of the adjacent LA and LV areas, therefore it works like a “sphincter” [2]. Pre-systolic contraction of the MA is related to LA contraction, and minimal MA area during early LV systole, suggesting that complete MA contraction requires both and properly timed LA and LV systole [13]. Contrariwise, changes in dimensions and function of the MA are accompanied with different disorders (cardiomyopathies, cardiac amyloidosis, ischaemic heart disease, etc.), which could influence LA and LV alone or via consecutive mitral regurgitation, as well [14]. Moreover, severe aortic valve stenosis is mostly accompanied with different remodeling patterns including concentric, mixed and dilated hypertrophy in response to pressure overload leading to MA abnormalities as well [15].

3DSTE seems to be an optimal imaging tool for simultaneous evaluation of LA volumes and two-dimensionally projected MA dimensions on a certain plane with respect to the cardiac cycle. Moreover, using (end-)systolic and (end-)diastolic data, several LA and MA functional properties could be calculated at the same time allowing evaluation of their relationship on each other [6]. 3DSTE is based on ‘block-matching algorhythm’, it was found to be suitable not only for chamber quantifications [4], but also for the assessment of valvular annular dimensions [9, 10].

The strong relationship between LA and MA dimensions and functions even in cases without known pathological states should highlight our attention on the fact, that any changes in LA volumes are accompanied with changes in MA dimensions possibly proceeding into functional valvular regurgitation.

Recent echocardiographic methods including measurement of strains offer significant potentials in the evaluation of LA function. Clinical usefulness and relevance of adding LA strain to LA volume index in the detection of LV diastolic dysfunction were found in patients with preserved LV-EF [16]. Moreover, LA function was found to be correlated with LV deformation [17]. These facts could highlight our attention on the relationship between LA strains and MA parameters, which could be a topic of future publications.

## 5. Limitation Section

- 2DE still have better image quality as compared to that of recently available 3DSTE systems, which could affect results [18]. The 3DSTE-derived acquisition allows 3D pyramidal echocardiographic full volume, but requires 4–6 cardiac cycles and gated capture to reconstruct the 3D image creating an opportunity for stitching artifacts. Moreover, rhythm disturbances and respiratory motion are also making imaging difficult. These facts could explain higher ratio of subjects who were excluded due to suboptimal image quality [19].

- Not the 3D saddle-shape of the MA, but its two-dimensionally projected image was assessed by 3DSTE analysis.

- Comparison of different echocardiographic modalities in the assessment of LA and MA dimensions was not aimed.

- Furthermore, validation of echocardiographic techniques was not purposed either due to their validated nature.

- No strain parameters featuring MA functionality was determined during the study.

- LA strains were not purposed to be determined [20].

- No volumetric or functional parameters including strains of both ventricles or the right atrium were assessed in this study.

- Fluid state of subjects were not controlled which could affect results.

- Differences could be demonstrated between published normal reference values of volumetric 3D echocardiography-derived LA volumetric data and 3DSTE-derived ones due to methodological differences.

## 6. Conclusions

3DSTE is suitable not only for chamber quantifications, but also for the assessment of valvular annular dimensions. Strong relationship exists between LA volumes and MA dimensions and functional properties in healthy subjects. With increasing LA volumes MA dilates and becomes functionally impaired which could explain developing functional mitral regurgitation in later stages.
